# Gamete signalling underlies the evolution of mating types and their number

**DOI:** 10.1098/rstb.2015.0531

**Published:** 2016-10-19

**Authors:** Zena Hadjivasiliou, Andrew Pomiankowski

**Affiliations:** 1 Centre for Mathematics and Physics in the Life Sciences and Experimental Biology, University College London, Gower Street, London WC1E 6BT, UK; 2 Department of Genetics, Evolution and Environment, University College London, Gower Street, London WC1E 6BT, UK

**Keywords:** sexes, mating types, isogamy, gametes, signalling

## Abstract

The gametes of unicellular eukaryotes are morphologically identical, but are nonetheless divided into distinct mating types. The number of mating types varies enormously and can reach several thousand, yet most species have only two. Why do morphologically identical gametes need to be differentiated into self-incompatible mating types, and why is two the most common number of mating types? In this work, we explore a neglected hypothesis that there is a need for asymmetric signalling interactions between mating partners. Our review shows that isogamous gametes always interact asymmetrically throughout sex and argue that this asymmetry is favoured because it enhances the efficiency of the mating process. We further develop a simple mathematical model that allows us to study the evolution of the number of mating types based on the strength of signalling interactions between gametes. Novel mating types have an advantage as they are compatible with all others and rarely meet their own type. But if existing mating types coevolve to have strong mutual interactions, this restricts the spread of novel types. Similarly, coevolution is likely to drive out less attractive mating types. These countervailing forces specify the number of mating types that are evolutionarily stable.

This article is part of the themed issue ‘Weird sex: the underappreciated diversity of sexual reproduction’.

## 1. Introduction: why have distinct mating types and how many?

While sexual reproduction requires two parents, there is no obvious need for them to be differentiated into distinct mating types or sexes. Yet, this is the predominate state of nature, from complex birds, mammals and plants down to humble single-celled eukaryotes. Sexual reproduction in complex organisms is contingent upon highly specialized male and female roles both at the organismal level (e.g. sex-specific attraction mechanisms) and cellular level (e.g. egg and sperm motility and size differences).

This picture changes when considering unicellular organisms. Although an asymmetry in gamete size (anisogamy) exists in some unicellular taxa, the vast majority of unicellular protist gametes are morphologically identical (isogamy) [1]. Yet the gametes of isogamous species are divided into genetically distinct mating types. These mate disassortatively, scarcely ever with members of the same type. This arrangement is paradoxical as it comes with a major cost since it restricts the pool of potential partners to those of a different mating type. Furthermore, this cost is maximized with two mating types, which perplexingly is the most common state in nature among isogamous organisms.

Considerable effort has been expended in forming and testing hypotheses to explain the evolution of mating types [2]. A prevalent explanation drawn from the literature on multicellular organisms suggest that mating types serve to avoid inbreeding by preventing matings between members of the same clone [3–6]. Another notable hypothesis proposes that mating types evolved because different gamete types can enforce uniparental inheritance of the cytoplasm, thereby restricting the spread of mitochondrial mutations or selfish elements [7–11]. Both are persuasive ideas but not without problems. The key challenge to both of them is the presence of several species where inbreeding and biparental inheritance is the rule but that nonetheless maintain mating types [12]. For example, in budding yeast the parent is diploid (heterozygous for the mating types alleles a and *α*) and undergoes meiosis to produce four haploid spores, two of each mating type. The spores then germinate and mate within the same tetrad while inheriting cytoplasm from both parents. Similar behaviour is encountered in a variety of other groups (e.g. *Neurospora tetrasperma, Gelasinospora tetrasperma, Podospora anserina and P. tetraspora* [13]).

We do not consider these theories further here, as they have been subject to several recent reviews [2,12,14]. Instead we focus and expand a neglected hypothesis first proposed by Hoekstra [15]. He suggested that mating types are determined by the molecular system regulating gamete interactions. The underlying idea is that partner recognition and pairing are more efficient when gametes produce recognition/attraction molecules and their receptors in a mating-type-specific manner. Indeed, in the absence of any asymmetry, cells will saturate their own receptors and compromise their ability to detect and find partners [12,16]. This is a compelling idea bringing cell–cell signalling to the centre of mating-type evolution.

The evolution of sexual signalling and mating preference has received great attention among multicellular organisms [17]. However, the same processes in the unicellular world have been barely addressed, particularly among isogamous species lacking obvious differentiation. This neglect in part reflects the popular assumption that opposite mating-type fusions exist for reasons unrelated to the signalling interaction itself (e.g. inbreeding avoidance and control of organelle inheritance, as discussed above). In addition, it is generally assumed that sex-specific roles follow from asymmetry in gamete morphology and motility (e.g. [18]), and this has overshadowed consideration of asymmetric signalling among isogamous species. To rectify this imbalance, we review signalling between gametes in isogamous species and show that asymmetry in gamete communication is universal. We argue that this asymmetry's primary function lies in promoting mating success. We then develop a simple model of gamete signalling and mating-type evolution that explains why the number of mating types is so frequently restricted to two and provides conditions under which more numerous mating types are favoured.

## 2. Review: gamete signalling

**Figure 1.**
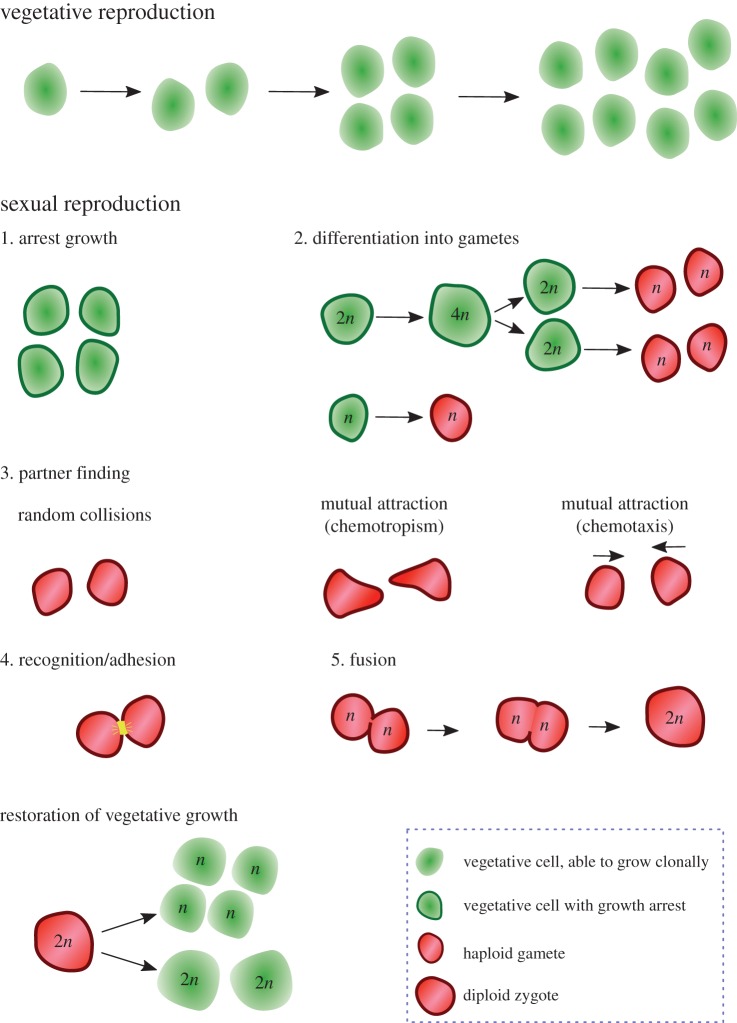
Model life cycle for unicellular eukaryotes. Cells grow vegetatively for as long as conditions allow. Entry into the sexual phase begins with growth arrest (1) followed by differentiation into gametes (2). Diplontic species undergo meiosis to produce haplontic gametes, whereas haploid species simply differentiate into sex-competent cells. Gametes or sex cells encounter one another (3), either by chance (e.g. *Chlamydomonas reinhardtii*), via directed growth following diffusible pheromones (e.g. yeasts) or through sexual chemotaxis (e.g. *Closterium*). When cells come in contact they recognize and adhere to one another (4). This is followed by cell and nuclear fusion (5). The diploid zygote then switches back to the vegetative programme in diplontic species or undergoes meiosis to produce haploid vegetative cells.

In unicellular organisms, sex is initiated when individuals (vegetative cells) are subject to growth arrest and produce sex-competent cells, either through meiosis (in diplontic species) or differentiation into gametes (in haplontic species; figure 1). This occurs as a response to environmental cues and/or substances released by other individuals of the same species. Following sexual differentiation, gametes must find and recognize other sex-competent cells of the same species, form adhesion and conjugation pairs, and synchronously permit fusion (figure 1). Here, we review gamete signalling interactions in unicellular and some multicellular species with isogamy, and point out the role of mating-type asymmetry.

### (a) Algae

#### (i) Chlamydomonas

**Figure 2.**
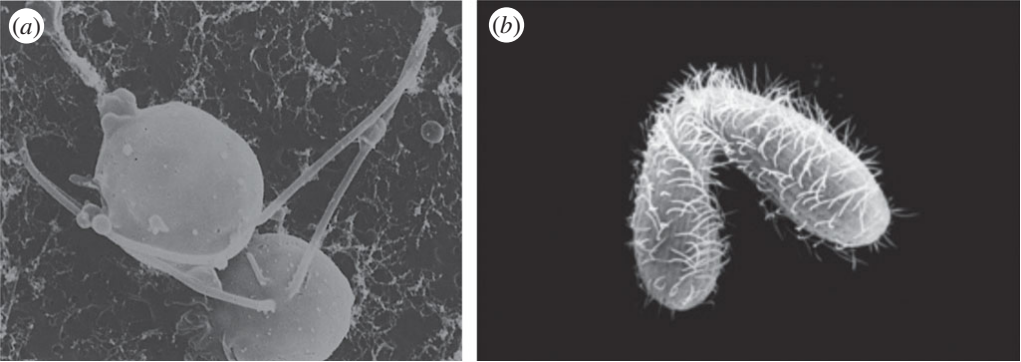
(*a*) Two *Chlamydomonas* cells undergoing fusion. Picture reproduced from Goodenough & Weiss [24] with permission from the authors. (*b*) *Tetrahymena* cells conjugating in preparation for nuclear exchange. Picture credit: SEPA ASSET programme at Cornell University.

*Chlamydomonas* species are biflagellate algae with two mating types (*MT*^+^ and *MT*^−^) and are generally isogamous. Haploid vegetative cells differentiate into gametes when environmental nitrogen levels drop [19]. The *MT* locus is located in a chromosomal region carrying several large inversions and translocations that suppress recombination. The *MT*^+^ and *MT*^−^ variants contain a number of genes that code for differentiation into + or – gametes, including mating-type-specific agglutinins that act as recognition and adhesion molecules. When gametes of the opposite mating type meet the agglutinins along their flagella interlink and adhere. Adhesion initiates a cascade that results in a 10-fold increase in intracellular cAMP, which enhances agglutinin levels and flagellar adhesiveness [20–22]. It also leads to the release of lytic enzymes that lead to rapid gamete cell wall disassembly and simultaneous production of complementary mating structures that prepare the gametes for fusion (figure 2*a*) [23–25]. Individual cells develop mating structures and fusion competence when exposed to conspecific substances even in the absence of a partner, pointing at the pivotal role of agglutinins in the mating process [25]. Following fusion, the two gametes contribute distinct information that is necessary for zygote development by forming heterodimers between the transcription factors Gsm1 and Gsp1 that are expressed differentially in the two mating types [19]. The heterodimers initiate zygote differentiation and meiosis. There is also evidence for sexual chemotaxis in some species of *Chlamydomonas*. At least one of the two mating types is attracted to substances released by the other type, but the putative substances have not been isolated or characterized [26,27].

#### (ii) Closterium

*Closterium* are diploid green algae and the closest unicellular relatives to land plants. Most *Closterium* species have two mating types, mt^+^ and mt^−^. Their sexual reproduction is well characterized and takes place in five steps: sexual cell division producing sexually competent cells (SCD), cell pairing, conjugation and papillae formation, protoplast release (i.e. loss of the cell wall) and protoplast fusion to produce a zygospore [28]. The presence of chemical substances responsible for coordinating sexual activity was postulated for *Closterium* species as early as 1971 [29]. It is now known that in the *Closterium peracerosum-strigosum-littorale* (*C. psl*) species complex, PR-IP (protoplast release-inducing protein) inducer is secreted by mt^−^ cells which stimulates SCD, protoplast release and mucilage secretion activity in mt^+^ cells [30,31]. The induction activity differs according to the PR-IP inducer concentration: low to mucilage secretion, medium to SCD and high to protoplast release in mt^+^ cells [28]. The same is true for the corresponding mt^+^ substance simulating the equivalent concentration-dependent reactions in mt^−^ cells. Similar multifunction mating type factors have been identified also in *Closterium ehrenbergii* [32,33]. In addition, mt^+^ and mt^−^ cells of *C. ehrenbergii* and *Closterium acerosum* migrate towards one another when separated [34–36]. This suggests the presence of mating-type-specific chemoattraction between opposite types, but these putative substances have not yet been characterized.

#### (iii) Diatoms

Diatoms have a unique diplontic vegetative phase involving size reduction associated with mitotic divisions [37]. The switch to sexual reproduction only occurs in cells below a critical size. Haploid gametes are generated via meiosis and are unable to grow clonally, so they must fuse to return to the diploid stage (or die). It has long been speculated that diatoms use pheromone signals to coordinate sexual reproduction [37]. Recent work has identified some of the components of this system in the pennate diatom *Seminavis robusta*. MT^+^ cells produce SIP^+^, a pheromone that induces cell cycle arrest and gamete production in MT^−^cells. It also induces proline biosynthesis and release of the pheromone diproline from MT^−^ cells. Diproline acts as a chemoattractant for MT^+^ cells [38,39]. The role of these reciprocal pheromones and other substances in subsequent stages of diatom mating (i.e. recognition and fusion) is not currently known.

#### (iv) Brown algae

Brown algae are multicellular marine algae. Sexual reproduction can be isogamous, anisogamous (different size gametes) or oogamous (large egg and small sperm). Pheromones in brown algae have been studied extensively and are well characterized in terms of their function and molecular composition [40]. In isogamous brown algae such as *Scytosiphon lomentaria*, *Colpomenia bullosa* and *Ectocarpus siliculosus*, the female-equivalent mating type releases pheromones that attract the male-equivalent mating type [41,42]. In *E. siliculosus*, mating-type-specific glycoproteins and receptors are responsible for gamete recognition and adhesion [42,43]. While the two mating types of *E*. *siliculosus* are morphologically the same, their mating behaviour is different. The + gametes swim for a short period of time after which they ingest their flagella and secrete pheromones. The − gametes, on the other hand, swim for prolonged periods and have pheromone receptor sites for signal processing necessary for their chemotactic response. They recognize the + gametes through receptors on their anterior flagellum, but the details of how this is achieved remain unexplored [43]. Transcriptome profiling of + and – gametes of *E. siliculosus* demonstrates extensive asymmetry between the two mating types highlighting that distinct sexual roles precede morphological differentiation of gametes [44].

### (b) Fungi

#### (i) Yeasts

Yeasts are isogamous, single-celled fungi, with two mating types. The vegetative stages of yeasts can be predominantly haploid (e.g. *Schizosaccharomyces pombe*) or diploid (e.g. *Saccharomyces cerevisiae*). Mating type is determined at the haploid level at a single genetic locus, *MAT*. The pertinent genes for each mating type are differentially expressed at this locus. The sexual cycles of yeasts begin with growth arrest and differentiation into gametes. This occurs as a response to environmental cues but, in addition, mating-type-specific pheromones initiate gametogenesis when sensed by the opposite type [45]. In both *S. pombe* and *S. cerevisiae*, binding to pheromone from the opposite mating type causes expression of mating-type-specific genes, and induces physiological and morphological changes leading to sexual differentiation [45]. Polarization of individual gametes along the pheromone gradient leads to directed growth towards gametes of the opposite mating type [45]. Mating-type-specific pheromones have similar functions in several other yeasts [46–49].

The molecular processes leading to gamete fusion are known in considerable detail [45]. It begins with the induction of mating-type-specific agglutinins by the opposite mating-type pheromone. The interlinking of agglutinins leads to cell adhesion [50,51] and increases conjugation efficiency [52]. Budding yeast mutants unable to produce mating pheromones cannot induce agglutination or conjugation and are effectively sterile [53]. Although exogenous pheromone restores agglutination, it does not lead to conjugation and fusion, suggesting that modulation of the pheromone concentration and the timing of secretion control downstream pathways crucial for mating [53]. As in *Chlamydomonas*, upon zygote formation transcription factors expressed differentially in the two mating types in yeasts form heterodimers that repress genes involved in mating and the haploid life cycle and are crucial for subsequent zygote development and meiosis [54,55].

#### (ii) Filamentous ascomycetes

Mating-type genes have also been studied in several filamentous ascomycetes. During sexual development, opposite mating types form male and female structures (defined as the donor and receiving structures, respectively) that fuse with one another allowing the transfer of the male nucleus to the female structure [56,57]. The entry of the male nucleus into the female hyphal cell stimulates fruiting body formation. The nuclei from the two mating types do not fuse but undergo repeated mitotic divisions in synchrony resulting in a fruiting body composed of cells with multiple nuclei from both mating types. The nuclei are then organized into dikaryotic cells, with one nucleus of each mating type, which fuse, and undergo meiosis and spore production.

The mating types regulate directed growth of the female structures towards the male, partner recognition, fertilization, fruiting body formation and nuclear coordination in the fruiting body [56,58,59]. The female trichogynes are attracted towards the male spermatia, suggestive of diffusible pheromones [60–62]. Pheromone precursor genes have been identified in many filamentous ascomycetes including *Cryphonectria parasitica* [63], *Magnaporthe grisea* [64], *Podospora anserina* [65] and *Neurospora crassa* [66]. The pheromone precursor genes are expressed in a mating-type-specific manner, similar to yeast pheromones. In some species such as *Ascobolus*, differentiated sexual structures only develop following opposite-mating-types interaction [67]. For example, the male element in *A. stercorarius* undergoes sexual activation after contact with the mycelium of the opposite mating type [68]. Other filamentous species like *P. arsenia* have the ability to differentiate sexual structures without mating-type interactions [62]. Interestingly, *P. arsenia* is a pseudohomothallic species, meaning that a single individual carries both mating types and so compatible partners are always present. This eliminates the need to have a check-point prior to sexual differentiation to ensure the presence of a partner.

A further key role of the mating types is the specification of nuclear identity, coordination of nuclear pairing and migration of nuclei into the dikaryotic hyphal cell [56]. In *P. anserina*, nuclear recognition is regulated by FMR1 and SMR2 proteins in the *mat^−^* nucleus and the FPR1 protein limited to the *mat*^+^ nucleus. When nuclei of the opposite mating type approach one another they release signals that simulate growth and nuclear migration, the success of this process relying on the proper association between the two nuclei [57,69]. Mutations in these genes affect nuclear synchronization, causing errors in dikaryote formation and barren fruiting bodies [70].

#### (iii) Filamentous basidiomycetes

Basidiomycetes spend most of their life cycle as dikaryons. Each cell holds two nuclei from the mating partners without fusion, and this state persists during the asexual phase [71]. Mating-type identity is determined by alleles at either one locus (bipolar system) or two unlinked loci (tetrapolar system). In the tetrapolar system, fusion normally only occurs between individuals that differ at both mating-type loci (e.g. *A1B1* × *A2B2*). Basidiomycetes are notable for having multiple mating types ranging from two up to several thousands, with multiple alleles possible at both loci. Mating types mediate pheromone signalling, cell fusion, filamentous growth in the dikaryote phase and preservation of compatible nuclei in close association through synchronized nuclear division [58,59,71].

Heterobasidiomycetes use pheromone signals to mediate mating partner choice, with pheromone interaction with receptors initiating the mating process when haploid isogamous cells or organs of the opposite mating type come in contact. *Ustilago hordei* has a bipolar mating system and two mating types. Mating-type pheromones in this species induce conjugation and tube formation in opposite mating types that grow chemotactically towards one another [72]. In the related *U. maydis*, following pheromone binding, mating structures are formed that enable fusion and the formation of the dikaryon. After fusion, mating-type alleles of the two partners form heterodimers that enter the diploid nucleus and control switching to filamentous growth and the subsequent meiosis [73,74]. Remarkably, these tight interactions occur despite *U*. *maydis* having some 50 mating types, which are determined at two multi-allelic loci [71].

In homobasidiomycetes (mushrooms), fusion between mycelia occurs independently of mating type. In these fungi, mating-type pheromones are activated following fusion and control formation of nuclear pairs in the dikaryon and maintain the dikaryophase [75]. A notable example is that of *Schizophyllum commune* that has thousands of mating types. Molecular analyses have found more than 75 different pheromones and several receptors [76]. Each distinct mating type consists of several genes specifying a single pheromone receptor pair [76]. But the receptors within a mating type never bind their own pheromones [77]. The pheromones and receptors control nuclear recognition and fusion within the mycelium [77,78]. A high degree of specificity is required for nuclear communication and the full completion of sexual development [76]. Although the mating system of *S. commune* restricts sibling matings to some extent, these are still possible approximately 25% of the time, suggesting that inbreeding avoidance cannot be the main function of these complex mating interactions. The situation is similar in other mushroom species such as *Coprinus cinereus* [79].

### (c) Amoebozoan slime moulds

In the cellular slime moulds, the unicellular phase of the life cycle is initiated following spore release from the fruiting body. The spores germinate and release haploid amoeboid cells that grow vegetatively while food supplies are abundant. Under stressful conditions, the unicellular amoebae either aggregate to form a new fruiting body or fuse to form a diploid zygote giant cell known as a macrocyst [80,81]. Macrocysts form through the fusion of cells with different mating types. In the well-studied *Dictyostelium discoideum*, there are three mating types, one of which appears to be a fusion of the other two [80]. At least two interacting mating-type-specific pheromones are necessary for macrocyst development and completion of the sexual phase [82]. Disruption of the mating-type genes suppresses cell fusion in *D. discoideum* [83,84]. However, the role of mating-type genes is poorly understood in this and other slime moulds such as *D. purpureum* and *D. giganteum* [85,86].

### (d) Ciliates

Mating in several ciliate groups does not involve cell fusion. Instead, compatible mating types form conjugating pairs, followed by exchange of nuclei through a conjugation bridge [87]. The conjugants then separate and the nuclei in each cell fuse before restoring the vegetative phase. Ciliates contain two nuclei, the micronucleus and the macronucleus. The diploid micronucleus undergoes meiosis. The ‘somatic’ macronucleus forms from massive rearrangement, amplification and gene loss from the diploid micronucleus.

The number of mating types in the genus *Euplotes* varies from five to 12 [87]. Many species, including *E. octocarinatus*, *E. raikovi*, *E. patella* and *E. woodru*, secrete mating-type-specific substances [87,88]. Individual cells grow vegetatively when binding to their own pheromone secreted continuously in the extracellular environment. Mature cells arrest growth and develop mating competence only when they bind to a non-self pheromone. The same substances also act as chemoattractants and sexualized cells are attracted to all non-self pheromones [89–91]. The interaction between mating-type pheromones in several ciliate species also regulates adhesion and conjugation between complementary gametes [91–93]. Some species of *Euplotes* reportedly do not secrete mating-type substances [87]. Instead, mating-type-specific interactions occur upon contact and prepare cells for conjugation [94]. It is worth noting that more recent reports suggest that *E. crassus,* a species previously thought to only carry surface-bound pheromones, actually does secrete pheromones but it remains to be seen whether diffusible pheromones are universal in *Euplotes* [95].

Cell adhesion is mediated through cilia binding via mating-type non-specific adhesins. However, mating-type-specific pheromones and receptors are used to coordinate adhesion and fusion. For example, the ciliate *Dileptus margaritifer* forms mating pairs due to the expression of mating-type non-specific cell-surface molecules [93]. The two partners coordinate the expression of their adhesion proteins by secreting and responding to pheromones in a mating-type-specific manner. Experiments found that conjugation is highly unstable between gametes of the same mating type [93]. It appears that continued stimulation using pheromones is needed until fusion is completed. Similar results were reported for *E. octocarinatus* where pairs of the same mating type were able to form under laboratory conditions but were unstable and generally separated before entering meiosis [96].

*Paramecium* is an exception among ciliates in that sexual cells produce mating-type-specific agglutinins [87]. In the isogamous *Paramecium bursaria,* mating-type-specific substances are responsible for pair formation, conjugation, adhesion and fusion [97]. The mating reaction following mixing of opposite mating types was observed in a number of different species [98], suggesting that similar substances coordinate mating in a number of different species of *Paramecium*. However, few details of the molecular signalling interactions are currently known. Finally, sexual chemotaxis does not occur among *Paramecia*. However, cell movement inactivation was reported following opposite-mating-type contact-mediated interactions [99].

## 3. The role of signalling asymmetry

Mating is contingent upon a cascade of events orchestrated between the mating cells. Our review of isogamous species reveals that these interactions are universally regulated by mating types, which produce a range of mating-type-specific proteins. These control a number of processes from initial sexual differentiation, through to gamete fusion and subsequent events in somatic development. The interactions between mating types are always asymmetric—a particular mating type will stimulate others (whether there are two or multiple) but always fails to stimulate cells carrying the same mating type. This asymmetry in intercellular signalling during sex appears to be fundamental to the evolution of mating types [12,16]. On what basis is asymmetry important? We address this question for each of the steps in the sexual cycle.

**Figure 3.**
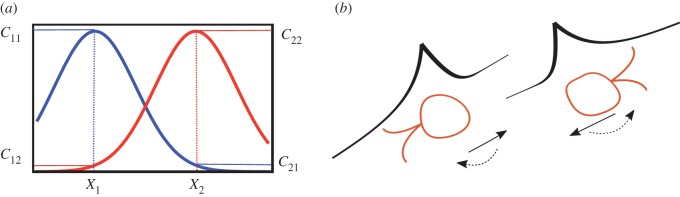
(*a*) The concentration profiles around two secreting cells centred at *X*_1_ and *X*_2_. The local concentration due to own pheromone *C*_11_ (blue) or *C*_22_ (red) is always higher than that of a remote cell *C*_12_ (red) or *C*_21_ (blue) at *X*_1_ or *X*_2_ respectively. A very high density of neighbouring cells would be required to generate concentration profiles that exceed those generated by an individual cell's own secretion. (*b*) Moving and pheromone sending generate a tail of high concentration behind moving secretors. This would prompt chemotactic cells that move towards one another to reverse their motion, unless they use distinct pheromones.

Diffusible signals are at least in part necessary for growth arrest and sexual differentiation in a range of species, including yeasts, ciliates and diatoms. Why cannot all cells send and differentiate in response to the same signal at this stage? Cells would then face the challenge of distinguishing their own signal from that of potential partners [12]. Owing to diffusion, signals from self will always be higher than those of a remote partner, considerably degrading the ability to distinguish self and other signals (figure 3*a*). Recent experiments have reinforced this idea by showing that secreting and detecting the same molecule can prevent cells from responding to signals from others, particularly at low cell densities [100]. In many species, differentiation into gametes is not reversible and the only way for individuals to restore their mitotic growth phase is by sexual fusion. It is therefore vital that passage into gametogenesis is synchronized with others, who are potential partners.

Following sexual differentiation individuals must pair. The majority of species reviewed here (with the exception of some species of ciliates and some chlamydomonads) use pheromones to direct migration or growth towards one another. Sending and receiving the same chemotactic signal could be problematic due to the potential of receptor saturation [15]. Experimental overexpression of pheromone disturbs gamete polarization in yeast gametes resulting in growth in a random direction, and a 15-fold increase in mating time [101]. An additional problem is that secretion during movement results in high chemoattractant concentration behind moving cells due to diffusion and accumulation of chemical molecules [16]. This alters the net local concentration, reducing the cell's ability to respond appropriately to external signals, or worse prompting the cell to reverse its direction of movement (figure 3*b*). Consequently, the use of chemotaxis to bring partners together likely provides a substantial advantage if attraction signals are sent and received asymmetrically [16].

Upon physical contact gametes must recognize one another as conspecifics, adhere and proceed to conjugation and/or fusion. There is strong selection for swift initial recognition when there is competition between gametes; for example, only two from an initial clump of several cells will mate in *Paramecium* and *Chlamydomonas* [19,102]. Furthermore, conjugation and fusion involve cell wall remodelling and must be tightly coordinated, as lack of synchronization can lead to osmotic shock and cell lysis [45]. Several species use the same mating-type pheromone/receptor pairs to induce gametogenesis as well as sexual chemotaxis. This is achieved by shifts in pheromone concentration inducing corresponding shifts in the other mating type's pheromone production. This would be difficult to achieve without asymmetric signals, as distinguishing changes in own versus partner pheromone production would be nigh on impossible (figure 3*a*). Several species use non-diffusible, surface-bound molecules for adhesion, conjugation and fusion, which are distinct from their pheromones and receptors. These again show mating-type specificity in species like *Paramecium* and some *Chlamydomonas*. A probable reason for this asymmetry lies in the avoidance of binding between self molecules that could lead, not only to saturation, but also to noise/interference that could potentially impair fusion synchronization or rapid cell–cell recognition. There is little specific theoretical work on this possibility beyond Hoekstra's original work [15], though cell surface interactions likely mirror the situation with diffusible signals and their receptors, and imposes a cost on the speed and robustness of the interaction when there is no asymmetry [16]. There is also a need for experimental work to investigate these trade-offs.

Finally, mating-type-specific molecules can also be important for post-fusion events. Heterodimers of proteins specific to each of the mating partners are important for switching the mating programme off and initiating meiosis in several species. It has been argued that this function is itself key to the evolution of mating types [12,103]. For example, it allows cells to assess their ploidy level and so switch between vegetative growth, and gametic developmental programmes, and between mitosis and meiosis. From our perspective, this is but one of the factors that favour asymmetry.

## 4. Pairwise gamete interactions dictate the number of mating types

Let us now assume that the key role of mating types lies in securing an asymmetry in signalling interactions between gametes. We expect a proliferation of mating types as new rare types seldom meet themselves and so have an advantage over more common types (i.e. negative frequency dependence), leading to their spread until they reach a frequency equal to that of the residents [2,104]. This appears to describe the case in some ciliates and basidiomycetes that have very large numbers of mating types. However, most known isogamous species have only two mating types. Clearly, there must be some constraints that operate on gamete signalling limiting the evolutionary proliferation of mating types. We investigate this in a simple model that considers the strength of pairwise interactions between gametes and how this affects the spread and elimination of new types.

### (a) Model outline

Consider a population with a single haploid locus *M* that defines the mating type, and assume *n* mating types are possible determined by alleles {*m*_1_, *m*_2_, … , *m_n_*}. We define the signalling preference of mating type *i* towards mating type *j* as *p_ij_* and let *p_ij_* take any positive value. This can be thought of as the ‘investment’ of mating type *i* in interactions with mating type *j*. Note, a more extensive treatment could be done by separating search, recognition and fusion functions, but for simplicity we consider these together, coded by a single locus. The mating probability between mating types *i* and *j* then depends on the product of their relative preferences for one another 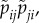 where 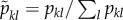.

This relative preference treatment can be justified by considering signalling systems such as those identified in many ciliates and basidiomycetes having multiple mating types. Here, multiple pheromones from different mating types compete with one another for the same receptor [88]. Likewise, in many species, multicell clumps form before pairs become isolated and proceed to zygote formation, suggesting that several cells compete to mate at the same time [19,102]. Therefore, if a receptor has a stronger interaction with one pheromone, it is likely that this denudes the strength of interaction with others. We assume *p_ij_* = 0 for all *i* = *j* so that homotypic pairings are not possible (i.e. mating types are already present), as discussed earlier in the article. The frequency of the *i*th mating type in the population (*f_i_*) then follows:
4.1
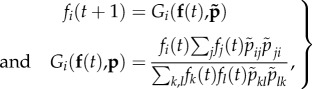
where the system of *n* equations with *f_i_*(*t* + 1) = *f_i_*(*t*) for *i* = 1, 2, … , *n* in equation (4.1) can be solved for the equilibrium value of each *f_i_*. This formulation follows from the assumption that the chance of successful mating between types *i* and *j* is proportional to their relative preferences and their frequencies in the population. The denominator in equation (4.1) is equivalent to the mean fitness and normalizes all frequencies at each time step. For *n* = 3, equation (4.1) simplifies to the following system of equations:
4.2
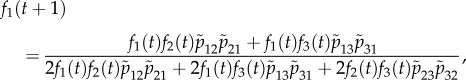

4.3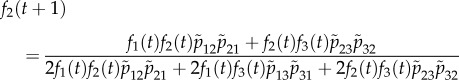 and 4.4
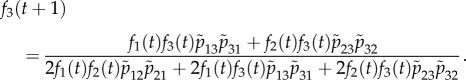


Solving these equations finds four equilibria for (*f*_1_, *f*_2_, *f*_3_) given by *E*_1_ = (0.5, 0.5, 0), *E*_2_ = (0.5, 0, 0.5) and *E*_3_ = (0, 0.5, 0.5) at which one mating type is lost and the others occur at equal frequencies, and 
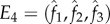
 where all mating types are present (i.e. 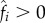 for all *i*). The value of each 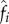 is a function of *p_ij_* given in the electronic supplementary material, equations S1–S3.

### (b) Model results

We begin by investigating the stability of *E*_1_, that is, when two mating types are resistant to invasion of a new mating type. If all preferences (*p_ij_*) are equal, then *E*_1_ is never stable, and a new mating type will always invade. Now consider the case where preferences vary. The full stability conditions are complex (given in the electronic supplementary material, equation S4), but can be simplified by assuming that *p*_12_ = *p*_21_ = *γ* (i.e. mating types 1 and 2 have similar preferences for each other). This is reasonable as we are assessing the condition when there are two mating types that have evolved to interact with one another, and their relationship is likely to be symmetric. We further assume that the rare new mating type 3 invests equally in interactions with either of the resident types, so *p*_31_ = *p*_32_ = *γ* + *η*, and that the two resident types have the same preference for the new mating type when it appears, so *p*_13_ = *p*_23_ = *γ* + *κ*. Under these assumptions, the stability condition for *E*_1_ reduces to
4.5
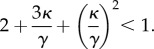


Both *η*, *κ* > –*γ*, because *p_ij_* has to always be positive, and condition (4.5) reduces to 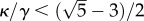 or 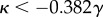 (see the electronic supplementary material for detailed derivation). A third rare type will therefore not invade if the interaction or preference of existing mating types towards it is about a third (38%) weaker than between the existing types. Similarly, if two mating types develop stronger preference for one another, while weakening their preference for a third type, the third type will be eliminated if it is 38% less preferred by the other two (electronic supplementary material, figure S1).

We also examined the more general situation when the preference of the resident types (types 1 and 2) for the new type (type 3) is not symmetric, by setting *p*_13_ = *γ* + *κ*_1_ and *p*_23_ = *γ* + *κ*_2_. The stability condition for *E*_1_ now reduces to
4.6
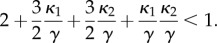


**Figure 4.**
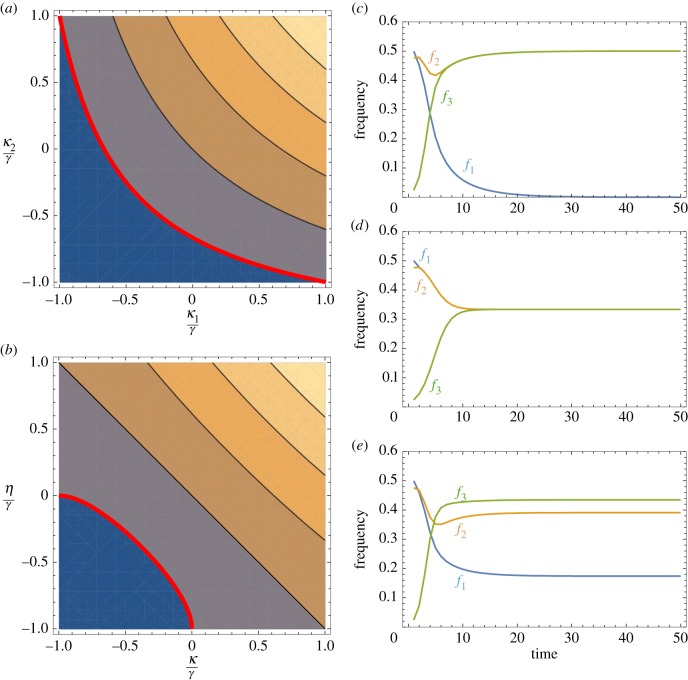
(*a,b*) Contour plots indicating stability conditions for *E*_1_. (*a*) Assuming that, *p*_12_ = *p*_21_ = γ, *p*_31_ = *p*_32_ = *γ* + *η*, *p*_13_ = *γ* + *κ*_1_ and *p*_23_ = *γ* + *κ*_2_ (condition (4.6) in main text) and (*b*) assuming that *p*_12_ = *p*_21_ = *γ*, *p*_12_ = *p*_31_ = *γ* + *η* and *p*_23_ = *p*_32_ = *γ* + *κ* (condition (4.7) in main text). In each instance, *E*_1_ is stable for values that lie below the red line. (*c–e*) Evolution of mating-type frequencies assuming three possible mating types (*n* = 3). The frequencies of the three mating types are iterated over time using equations (4.2)–(4.4) in the main text. Values for *p_ij_* used: (*c*) *p*_12_ = 0.9, *p*_21_ = 0.6, *p*_31_ = 0.5, *p_ij_* = 1 for all other pairs (*i*,*j*), (*d*) *p_ij_* = 0.5 for all (*i*,*j*), (*e*) *p*_21_ = 0.5, *p_ij_* = 1 for all other (*i*,*j*).

In this case, a third type will not invade if larger values of *κ*_1_ are compensated by smaller values for *κ*_2_ and vice versa (figure 4*a*). Note that owing to the assumption that *p*_31_ = *p*_32_, *η* does not impact on the stability conditions (4.5) or (4.6).

An alternative treatment to simplify stability condition (S4) of the electronic supplementary material is to assume that all pairwise interactions occur symmetrically so that *p*_12_ = *p*_21_ = *γ*, *p*_12_ = *p*_31_ = *γ* + *η* and *p*_23_ = *p*_32_ = *γ* + *κ*. Now the stability condition for *E*_1_ reduces to
4.7
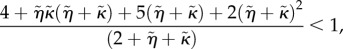
where 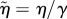 and 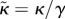. Condition (4.7) is illustrated in figure 4*b*. This shows that the stability condition for *E*_1_ holds if both 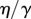 and 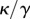 are negative. Once again, a third mating type cannot be established if the pairwise interaction (or preference) between the residents is sufficiently stronger than with the mutant. Conversely, a third mating type can replace one of the residents if it establishes strong interactions with the other one (figure 4*c*), or can invade at the expense of both mating types so that a polymorphic equilibrium with three mating types at equal or unequal frequencies is established (figure 4*d,e*).

We also explored more complex situations with four or more mating types. There are then multiple equilibria with variable numbers of mating types persisting. Although the analysis becomes more complex, the same principles hold. A stability analysis and simulations for *n* = 4 are provided in the electronic supplementary material.

Our model suggests that a rare type only increases in frequency if it is able to strongly interact with the pre-existing mating types (figure 4). It challenges the general belief that rare mating types invade as they have high mating rates because they rarely meet themselves [2]. This ignores the strength of interaction between mating types. We expect that if two mating types have coevolved for some time they will have gained strong interactions, say because this leads to more efficient mating. Further novel mating types lacking this strength of interaction thus will be less likely to invade. Likewise, in a population with three mating types one may become extinct if two types evolve significantly stronger interactions with one another. Underlying this are likely to be biochemical constraints on signal and recognition molecules, so the evolution of stronger interactions (higher preference) for one signal automatically weakens interactions with other mating types. Although such bias needs to be strong for other types to be eliminated altogether, it seems likely that small differences could seed subsequent coevolution, and drive the population towards fewer types. These concerns place major limitations on mating-type proliferation. They also suggest that where multiple mating types occur, such as in many ciliates and filamentous fungi, new forms must be equally able to interact with the existing types (or nearly so). It would be interesting to understand what structural properties of signals and receptors in these cases allow for this outcome.

## 5. Conclusion

The evolution of mating types and the optimal number of mating types remain key unanswered questions in evolutionary biology. In this work, we examined the capacity for asymmetric gamete signalling to address both of these apparent enigmas. This idea goes back to the pioneering work by Hoekstra in 1982 [15]. In support of this theory, we present evidence that asymmetric communication between opposite mating types is common, if not universal, throughout sexual reproduction in species with isogamous gametes. In addition, we discuss the constraints gametes encounter without any asymmetry in their signalling communication, beyond those postulated initially [12,15,16].

The evolutionary forces that determine the number of mating types have received even less attention than the evolution of mating types themselves. A pioneering study tackling this question suggests that selection for quick mating favours the evolution of multiple mating types [105]. Under this condition, novel mating types are at an advantage when rare as they can mate with more individuals they encounter than the existing more common types. It seems likely that gametes are generally under strong selection for quick mating, especially in unicellular species. But if this is the case, why are two mating types the dominant reproductive mode? It has also been suggested that mating types act as a self-incompatibility (SI) system that prevents inbreeding, and that negative frequency-dependent selection favours rare types as they can mate with more partners [3,4,6]. Furthermore, the allelic diversity and recombination between different loci that specify SI may also be important for the invasion of new mating types [106]. While these considerations might explain expansion in the number of mating types, they again fail to establish why two is the dominant reproductive mode. An alternative approach suggests that selection for uniparental inheritance of mitochondria (and other cytoplasmic genes like chloroplasts) requires two distinct mating types, one to be the transmitting sex and the other to destroy its mitochondria [104]. While there may be advantages to the uniparental inheritance of mitochondria, analyses of models allowing coevolution with mating types do not result in the stabilization of two mating types [8]. This hypothesis does not fully account for the proliferation of mating types and why this is restricted to certain groups.

We propose an alternative hypothesis based on the strength of pairwise interactions between gametes. In the model, novel mating types only spread if they interact strongly with the resident mating types. Spread is resisted if the existing mating types have evolved strong pairwise interactions that limit the attraction/recognition of novel variants. Conversely, multiple mating types evolve if specialization does not restrict interactions with novel types. The model also explains the observation that in species with multiple mating types not all types coexist at equal frequencies [107], as uneven pairwise interaction strengths lead to multiple mating types at different frequencies (figure 4*e*). Such unevenness could also arise due to drift in the vegetative growth phase, but then would be expected to be randomly distributed across mating types through time [6]. Our alternative model is not at odds with previous hypotheses, for example, selection for swift partner finding, SI or the need for uniparental inheritance of mitochondria [3,4,104,105]. However, it does identify a generally applicable selective force that may better account for variation in number and distribution of mating types seen in unicellular organisms.

The main challenge to the ideas presented here is the existence of homothallic species, where clones of the same mating type can mate with one another [2]. In many reported cases of homothallism, however, mating partners behave asymmetrically [108,109]. These behaviours could be due to mechanisms similar to mating type switching whereby clonal individuals express opposite mating types [6]. It would be interesting to assess whether asymmetries actually underlie the mating behaviour of gametes in homothallic species, and to quantify the efficiency of mating compared to related heterothallic species.

## Supplementary Material

Supplementary material
