# Supplementary material for: Gamete signalling underlies the evolution of mating types and their number
Source: Philos Trans R Soc Lond B Biol Sci. 2016 Oct 19;371(1706):20150531. doi: 10.1098/rstb.2015.0531 (PMC5031616; doi:10.1098/rstb.2015.0531)
Supplement: Supplementary material [file rstb20150531supp1.pdf]

Phil. Trans. R. Soc. B 2016. doi: 10.1098/rstb.2015.0531

Gamete signalling underlies the evolution of two and multiple sexes: Supplemental Information

Zena Hadjivasiliou[1,2], Andrew Pomiankowski[1,2]

1) CoMPLEX, Centre for Mathematics and Physics in the Life sciences and Experimental biology, University College London, Gower Street, London, UK. 2) Department of Genetics, Evolution and Environment, University College London, Gower Street, London, UK

1 Mathematical derivations

Equations (2 - 4) in the main text can be solved by setting $f_i(t+1) = f_i(t)$ for $i = 1,2,3$. This gives three equilibria:

$$E_1\colon (f_1, f_2, f_3) = (\tfrac{1}{2}, \tfrac{1}{2}, 0)$$

$$E_2\colon (f_1, f_2, f_3) = (\tfrac{1}{2}, 0, \tfrac{1}{2})$$

$$E_3\colon (f_1, f_2, f_3) = (0, \tfrac{1}{2}, \tfrac{1}{2})$$

$$E_4\colon (f_1, f_2, f_3) = (f_1^*, f_2^*, f_3^*)$$

where the expressions for f_1^*, f_2^*, f_3^* are given by,

$$f_1^* = \frac{p_{23}p_{32}(p_{12}+p_{13})(p_{13}(p_{23}(p_{32}-p_{31})-p_{21}p_{31})-p_{12}(p_{21}(p_{31}+p_{32})-p_{23}p_{32}))}{D} \tag{S1}$$

$$f_2^* = \frac{p_{13}p_{31}(p_{21}+p_{23})(p_{13}(p_{23}(p_{31}-p_{32})+p_{21}p_{31})-p_{12}(p_{21}(p_{31}+p_{32})+p_{23}p_{32}))}{D} \tag{S2}$$

$$f_3^* = \frac{p_{12}p_{21}(p_{31}+p_{32})(p_{12}(p_{21}(p_{31}+p_{32})-p_{23}p_{32})-p_{13}(p_{23}(p_{31}+p_{32})+p_{21}p_{31}))}{D} \tag{S3}$$

and the denominator D is given by,

$D = p_{13}^2(p_{21}p_{31}+p_{23}(p_{31}-p_{32}))^2+p_{12}^2(p_{23}p_{32}-p_{21}(p_{31}+p_{32}))^2-2p_{12}p_{13}(p_{23}^2(p_{31}-p_{32})p_{32}+p_{21}^2p_{31}(p_{31}+p_{32})+p_{21}p_{23}(p_{31}^2+3p_{31}p_{32}+p_{32}^2))$.

The Jacobian matrix of the three dimensional system ($n = 3$) evaluated at E_1 reads,

$$\begin{pmatrix} \frac{\partial G_1}{\partial f_1} & \frac{\partial G_1}{\partial f_2} & \frac{\partial G_1}{\partial f_3} \\ \frac{\partial G_2}{\partial f_1} & \frac{\partial G_2}{\partial f_2} & \frac{\partial G_2}{\partial f_3} \\ \frac{\partial G_3}{\partial f_1} & \frac{\partial G_3}{\partial f_2} & \frac{\partial G_3}{\partial f_3} \end{pmatrix}_{\left(\frac{1}{2},\frac{1}{2},0\right)} =$$

$$\begin{pmatrix} 0 & 0 & \frac{p_{31}(p_{12}+p_{13})(p_{21}+p_{23})\left(1-\frac{p_{12}}{p_{12}+p_{13}}\right)}{p_{12}p_{21}(p_{31}+p_{32})} - \frac{(p_{12}+p_{13})(p_{21}+p_{23})\left(\frac{\left(1-\frac{p_{12}}{p_{12}+p_{13}}\right)p_{31}}{p_{31}+p_{32}}+\left(1-\frac{p_{21}}{p_{21}+p_{23}}\right)\left(1-\frac{p_{31}}{p_{31}+p_{32}}\right)\right)}{p_{12}p_{21}} \\ 0 & 0 & \frac{(p_{12}+p_{13})(p_{21}+p_{23})\left(1-\frac{p_{12}}{p_{21}+p_{23}}\right)\left(1-\frac{p_{31}}{p_{31}+p_{32}}\right)}{p_{12}p_{21}} - \frac{(p_{12}+p_{13})(p_{21}+p_{23})\left(\frac{\left(1-\frac{p_{12}}{p_{12}+p_{13}}\right)p_{31}}{p_{31}+p_{32}}+\left(1-\frac{p_{21}}{p_{21}+p_{23}}\right)\left(1-\frac{p_{31}}{p_{31}+p_{32}}\right)\right)}{p_{12}p_{21}} \\ 0 & 0 & \frac{(p_{12}+p_{13})(p_{21}+p_{23})\left(\frac{\left(1-\frac{p_{12}}{p_{12}+p_{13}}\right)p_{31}}{p_{31}+p_{32}}+\left(1-\frac{p_{21}}{p_{21}+p_{23}}\right)\left(1-\frac{p_{31}}{p_{31}+p_{32}}\right)\right)}{p_{12}p_{21}} \end{pmatrix}$$

The eigenvectors of the Jacobian at (1/2, 1/2, 0) are, $\lambda_{1,2} = 0$ and $\lambda_3 = \frac{p_{13}p_{31}(p_{21}+p_{23})+p_{23}p_{32}(p_{12}+p_{13})}{p_{12}p_{21}(p_{31}+p_{32})}$. E_1 is stable iff $|\lambda_i| < 1$ for $i = 1,2,3$ and so the stability condition for E_1 reduces to,

$$p_{13}p_{31}(p_{21}+p_{23})+p_{23}p_{32}(p_{12}+p_{13}) < p_{12}p_{21}(p_{31}+p_{32}). \tag{S4}$$

Setting $p_{12} = p_{21} = \gamma$, $p_{31} = p_{32} = \gamma+\eta$ and $p_{13} = p_{23} = \gamma+\kappa$ in (S4) and rearranging we obtain,

$$2+3\kappa/\gamma+(\kappa/\gamma)^2 < 1$$

which is expression (5) in the main text. Similarly, setting $p_{12} = p_{21} = \gamma$, $p_{31} = p_{32} = \gamma+\eta$, $p13 = \gamma+\kappa 1$and $p23 = \gamma+\kappa 2$ in (S4) and rearranging we obtain,

$$2+\frac{3}{2}\frac{\kappa_1}{\gamma}+\frac{3}{2}\frac{\kappa_2}{\gamma}++\frac{\kappa_1}{\gamma}\frac{\kappa_2}{\gamma}<1$$

which is expression (6) in the main text. Finally, setting $p_{12}=p_{21}=\gamma$, $p_{13}=p_{31}=\gamma+\eta$ and $p_{23}=p_{32}=\gamma+\kappa$ in (S4), dividing through by γ^3 and rearrnaging we obtain,

$$\frac{4+\frac{\eta}{\gamma}\frac{\kappa}{\gamma}\left(\frac{\eta}{\gamma}+\frac{\kappa}{\gamma}\right)+5\left(\frac{\eta}{\gamma}+\frac{\kappa}{\gamma}\right)+2\left(\frac{\eta}{\gamma}+\frac{\kappa}{\gamma}\right)^2}{\left(2+\frac{\eta}{\gamma}+\frac{\kappa}{\gamma}\right)}<1$$

Setting $\tilde{\eta}=\frac{\eta}{\gamma}$ and $\tilde{\kappa}=\frac{\kappa}{\gamma}$ we the last expression reduced to expression (7) in the main text.

In order to obtain conditions under which $f_i^*=\frac{1}{3}$ for all i, we set $f_1^*=f_2^*=f_2^*=\frac{1}{3}$ in (S1-S3). These equations hold true if the following expressions linking p_{ij} are true,

$$p_{23}=\frac{p_{12}p_{21}}{p_{13}},p_{31}=\frac{p_{12}p_{32}}{p_{13}} \tag{S5}$$

or

$$p_{23}=-\frac{p_{13}p_{21}}{p_{12}+p_{13}},p_{31}=-\frac{(p_{12}+p_{13})p_{32}}{p_{12}} \tag{S6}$$

Since p_{ij} can only take positive, $f_i^*=\frac{1}{3}$ for $i=1,2,3$ iff $p_{23}=\frac{p_{12}p_{21}}{p_{13}}$ and $p_{31}=\frac{p_{12}p_{32}}{p_{13}}$. The Jacobian matrix under these assumptions is given by,

$$\begin{pmatrix} \frac{1}{3} & -\frac{1}{6} & -\frac{1}{6} \\ -\frac{1}{6} & \frac{1}{3} & -\frac{1}{6} \\ -\frac{1}{6} & -\frac{1}{6} & \frac{1}{3} \end{pmatrix}$$

and has eigenvalues $\lambda_{1,2,3}=0,\frac{1}{2},\frac{1}{2}$. It follows that if condition (S5) holds, then solution to Equations (2-4) in the main text is given by $f_i^*=\frac{1}{3}$ for $i=1,2,3$ all and is stable. We therefore expect three mating types at equal frequencies to be stable under these conditions.

2 Analysis with $n = 4$

In this section we expand our analysis to four dimensions, i.e. assuming four possible mating types. The frequencies of each mating type f_1, f_2, f_3, f_4 are described by the following system of equations,

$$\begin{aligned} f_1(t+1) &= (f_1(t)f_2(t)\tilde{p}_{12}\tilde{p}_{21} + f_1(t)f_3(t)\tilde{p}_{13}\tilde{p}_{31} + f_1(t)f_4(t)\tilde{p}_{14}\tilde{p}_{41})/ \\ &\quad (2f_1(t)f_2(t)\tilde{p}_{12}\tilde{p}_{21} + 2f_1(t)f_3(t)\tilde{p}_{13}\tilde{p}_{31} + 2f_1(t)f_4(t)\tilde{p}_{14}\tilde{p}_{41} + \\ &\quad 2f_2(t)f_3(t)\tilde{p}_{23}\tilde{p}_{32} + 2f_2(t)f_4(t)\tilde{p}_{24}\tilde{p}_{42} + 2f_3(t)f_4(t)\tilde{p}_{34}\tilde{p}_{43}) \end{aligned}$$

$$\begin{aligned} f_2(t+1) &= (f_1(t)f_2(t)\tilde{p}_{12}\tilde{p}_{21} + f_2(t)f_3(t)\tilde{p}_{23}\tilde{p}_{32} + f_2(t)f_4(t)\tilde{p}_{24}\tilde{p}_{42})/ \\ &\quad (2f_1(t)f_2(t)\tilde{p}_{12}\tilde{p}_{21} + 2f_1(t)f_3(t)\tilde{p}_{13}\tilde{p}_{31} + 2f_1(t)f_4(t)\tilde{p}_{14}\tilde{p}_{41} + \\ &\quad 2f_2(t)f_3(t)\tilde{p}_{23}\tilde{p}_{32} + 2f_2(t)f_4(t)\tilde{p}_{24}\tilde{p}_{42} + 2f_3(t)f_4(t)\tilde{p}_{34}\tilde{p}_{43}) \end{aligned}$$

$$\begin{aligned} f_3(t+1) &= (f_1(t)f_3(t)\tilde{p}_{13}\tilde{p}_{31} + f_3(t)f_2(t)\tilde{p}_{32}\tilde{p}_{23} + f_3(t)f_4(t)\tilde{p}_{34}\tilde{p}_{43})/ \\ &\quad (2f_1(t)f_2(t)\tilde{p}_{12}\tilde{p}_{21} + 2f_1(t)f_3(t)\tilde{p}_{13}\tilde{p}_{31} + 2f_1(t)f_4(t)\tilde{p}_{14}\tilde{p}_{41} + \\ &\quad 2f_2(t)f_3(t)\tilde{p}_{23}\tilde{p}_{32} + 2f_2(t)f_4(t)\tilde{p}_{24}\tilde{p}_{42} + 2f_3(t)f_4(t)\tilde{p}_{34}\tilde{p}_{43}) \end{aligned}$$

$$
\begin{aligned}
f_4(t+1) &= (f_1(t)f_4(t)\tilde{p}_{14}\tilde{p}_{41} + f_4(t)f_2(t)\tilde{p}_{42}\tilde{p}_{24} + f_4(t)f_3(t)\tilde{p}_{43}\tilde{p}_{34})\,/ \\
&\quad (2f_1(t)f_2(t)\tilde{p}_{12}\tilde{p}_{21} + 2f_1(t)f_3(t)\tilde{p}_{13}\tilde{p}_{31} + 2f_1(t)f_4(t)\tilde{p}_{14}\tilde{p}_{41} + \\
&\quad 2f_2(t)f_3(t)\tilde{p}_{23}\tilde{p}_{32} + 2f_2(t)f_4(t)\tilde{p}_{24}\tilde{p}_{42} + 2f_3(t)f_4(t)\tilde{p}_{34}\tilde{p}_{43})
\end{aligned}
$$

We set $f_i(t+1) = f_i(t)$ for all i in the equations above to solve the system. This gives 11 possible equilibria,

$$E_1 : (f_1, f_2, f_3, f_4) = (\frac{1}{2}, \frac{1}{2}, 0, 0)$$

$$E_2 : (f_1, f_2, f_3, f_4) = (\frac{1}{2}, 0, \frac{1}{2}, 0)$$

$$E_3 : (f_1, f_2, f_3, f_4) = (\frac{1}{2}, 0, 0\frac{1}{2},)$$

$$E_4 : (f_1, f_2, f_3, f_4) = (0, \frac{1}{2}, \frac{1}{2}, 0)$$

$$E_5 : (f_1, f_2, f_3, f_4) = (0, \frac{1}{2}, 0, \frac{1}{2})$$

$$E_6 : (f_1, f_2, f_3, f_4) = (0, 0\frac{1}{2}, \frac{1}{2})$$

$$E_7 : (f_1, f_2, f_3, f_4) = (f_1^7, f_2^7, f_3^7, 0)$$

$$E_8 : (f_1, f_2, f_3, f_4) = (f_1^8, f_2^8, 0, f_4^8)$$

$$E_9 : (f_1, f_2, f_3, f_4) = (f_1^9, 0, f_3^9, f_4^9)$$

$$E_{10} : (f_1, f_2, f_3, f_4) = (0, f_2^{10}, f_3^{10}, f_4^{10})$$

$$E_{11} : (f_1, f_2, f_3, f_4) = (f_1^{11}, f_2^{11}, f_3^{11}, f_4^{11})$$

where,

$$f_1^7 = \frac{p_{23}p_{32}(p_{12}+p_{13}+p_{14})(p_{14}p_{23}p_{32}-p_{13}(p_{21}p_{31}+p_{23}p_{31}+p_{24}p_{31}-p_{23}p_{32})-p_{12}(p_{21}(p_{31}+p_{32}+p_{34})-p_{23}p_{32}))}{D_7}$$

$$f_2^7 = \frac{p_{13}p_{31}(p_{21}+p_{23}+p_{24})(p_{13}(p_{21}p_{31}+p_{23}p_{31}+p_{24}p_{31}-p_{23}p_{32})-p_{14}p_{23}p_{32}-p_{12}(p_{23}p_{32}+p_{21}(p_{31}+p_{32}+p_{34})))}{D_7}$$

$$f_3^7 = \frac{p_{12}p_{21}(p_{31}+p_{32}+p_{34})(p_{12}(-p_{23}p_{32}+p_{21}(p_{31}+p_{32}+p_{34}))-p_{14}p_{23}p_{32}-p_{13}(p_{21}p_{31}+p_{24}p_{31}+p_{23}(p_{31}+p_{32})))}{D_7}$$

$$f_1^8 = \frac{(p_{12}+p_{13}+p_{14})p_{24}p_{42}(p_{13}p_{24}p_{42}-p_{14}(p_{21}p_{41}+p_{23}p_{41}+p_{24}p_{41}-p_{24}p_{42})-p_{12}(p_{21}(p_{41}+p_{42}+p_{43})-p_{24}p_{42}))}{D_8}$$

$$f_2^8 = \frac{p_{14}(p_{21}+p_{23}+p_{24})p_{41}(p_{14}(p_{21}p_{41}+p_{23}p_{41}+p_{24}p_{41}-p_{24}p_{42})-p_{13}p_{24}p_{42}-p_{12}(p_{24}p_{42}+p_{21}(p_{41}+p_{42}+p_{43})))}{D_8}$$

$$f_4^8 = \frac{p_{12}p_{21}(p_{41}+p_{42}+p_{43})(p_{24}(p_{41}+p_{42})-p_{13}p_{24}p_{42}-p_{14}(p_{21}p_{41}+p_{23}p_{41})+p_{12}(p_{21}(p_{41}+p_{42}+p_{43})-p_{24}p_{42}))}{D_8}$$

$$f_1^9 = \frac{p_{34}p_{43}(p_{12}+p_{13}+p_{14})(p_{12}p_{34}p_{43}-p_{14}(p_{31}p_{41}+p_{32}p_{41}+p_{34}p_{41}-p_{34}p_{43})-p_{13}(p_{31}(p_{41}+p_{42}+p_{43})-p_{34}p_{43}))}{D_9}$$

$$f_3^9 = \frac{p_{14}p_{42}(p_{31}+p_{32}+p_{34})(p_{14}(p_{31}p_{41}+p_{32}p_{41}+p_{34}p_{41}-p_{34}p_{43})-p_{12}p_{34}p_{43}-p_{13}(p_{34}p_{43}+p_{31}(p_{41}+p_{42}+p_{43})))}{D_9}$$

$$f_4^9 = \frac{p_{13}p_{31}(p_{41}+p_{42}+p_{43})(p_{34}(p_{41}+p_{43})-p_{12}p_{34}p_{43}-p_{14}(p_{31}p_{41}+p_{32}p_{41})+p_{13}(p_{31}(p_{41}+p_{42}+p_{43})-p_{34}p_{43}))}{D_9}$$

$$f_2^{10} = \frac{p_{34}p_{43}(p_{21}+p_{23}+p_{24})(p_{21}p_{34}p_{43}-p_{24}(p_{31}p_{42}+p_{32}p_{42}+p_{34}p_{42}-p_{34}p_{43})-p_{23}(p_{32}(p_{41}+p_{42}+p_{43})-p_{34}p_{43}))}{D_{10}}$$

$$f_3^{10} = \frac{p_{24}p_{42}(p_{31}+p_{32}+p_{34})(p_{24}(p_{31}p_{42}+p_{32}p_{42}+p_{34}p_{42}-p_{34}p_{43})-p_{21}p_{34}p_{43}-p_{23}(p_{34}p_{43}+p_{32}(p_{41}+p_{42}+p_{43})))}{D_{10}}$$

$$f_4^{10} = \frac{p_{23}p_{32}(p_{41}+p_{42}+p_{43})(p_{34}(p_{42}+p_{43})-p_{21}p_{34}p_{43}-p_{24}(p_{31}p_{42}+p_{32}p_{42})+p_{23}(p_{32}(p_{41}+p_{42}+p_{43})-p_{34}p_{43}))}{D_{10}}$$

$$
\begin{aligned}
f_1^{11} \;=\; & (p_{12}^2 p_{34} p_{43}(2p_{23}p_{24}p_{32}p_{42} + p_{21}^2 p_{34}p_{43} - p_{21}(p_{24}(p_{31}p_{42} + p_{32}p_{42} + p_{34}p_{42} - p_{34}p_{43}) + \\
& p_{23}(-p_{34}p_{43} + p_{32}(p_{41} + p_{42} + p_{43})))) - \\
& (p_{!3} + p_{14})(p_{14}p_{23}p_{32}(p_{21}p_{34}p_{41}p_{43} \\
& p_{24}(p_{31}p_{41}p_{42} + p_{32}p_{41}p_{42} + p_{34}p_{41}p_{42} + p_{34}p_{41}p_{43} - 2p_{34}p_{42}p_{43}) - \\
& p_{23}p_{41}(-p_{34}p_{43} + p_{32}(p_{41} + p_{42} + p_{43}))) + \\
& p_{!3}p_{24}p_{42}(-p_{31}(-p_{21}p_{34}p_{43} + p_{24}(p_{31}p_{42} + p_{32}p_{42} + p_{34}p_{42} - p_{34}p_{43})) + \\
& p_{23}(-2p_{32}p_{34}p_{43} + p_{31}(p_{34}p_{43} + p_{32}(p_{41} + p_{42} + p_{43}))))) + \\
& p_{12}(p_{14}(p_{23}^2 p_{32}p_{41}(-p_{34}p_{43} + p_{32}(p_{41} + p_{42} + p_{43})) \\
& +p_{21}p_{34}p_{43}(p_{21}p_{34}p_{43} - p_{24}(p_{31}p_{42} + p_{32}p_{42} + p_{34}p_{42} - p_{34}p_{43})) - \\
& p_{23}(p_{24}p_{32}(p_{31}p_{41}p_{42} + p_{32}p_{41}p_{42} + p_{34}p_{41}p_{42} + p_{34}p_{41}p_{43} - 4p_{34}p_{42}p_{43}) + \\
& p_{21}p_{34}p_{43}(-p_{34}p_{43} + p_{32}(2p_{41} + p_{42} + p_{43})))) \\
& -p_{13}(-(p_{24}p_{31}p_{42} - p_{21}p_{34}p_{43})(-p_{21}p_{34}p_{43} + p_{24}(p_{31}p_{42} + p_{32}p_{42} + p_{34}p_{42} - p_{34}p_{43})) + \\
& p_{23}(p_{21}p_{34}p_{43}(-p_{34}p_{43} + p_{32}(p_{41} + p_{42} + p_{43})) + \\
& p_{24}p_{42}(-4p_{32}p_{34}p_{43} + p_{31}(p_{34}p_{43} + p_{32}(p_{41} + p_{42} + p_{43}))))))))/D_{11}
\end{aligned}
$$

$$
\begin{aligned}
f_2^{11} \;=\; & ((p_{21} + p_{23} + p_{24})(p_{13}^2 p_{24}p_{31}p_{42}(-p_{34}p_{43} + p_{31}(p_{41} + p_{42} + p_{43})) + \\
& (p_{14}p_{23}p_{32}p_{41} - p_{12}p_{21}p_{34}p_{43})(-p_{12}p_{34}p_{43} + p_{14}(p_{31}p_{41} + p_{32}p_{41} + p_{34}p_{41} - p_{34}p_{43})) - \\
& p_{!3}(p_{12}p_{34}p_{43}(p_{24}p_{31}p_{42} + p_{21}(-p_{34}p_{43} + p_{31}(p_{41} + p_{42} + p_{43}))) + \\
& p_{14}(p_{31}(-2p_{21}p_{34}p_{41}p_{43} + p_{24}(p_{31}p_{41}p_{42} + p_{32}p_{41}p_{42} + p_{34}p_{41}p_{42} - 2p_{34}p_{41}p_{43} + p_{34}p_{42}p_{43})) \\
& +p_{23}p_{41}(p_{32}p_{34}p_{43} + p_{31}(-2p_{34}p_{43} + p_{32}(p_{41} + p_{42} + p_{43})))))))/D_{11}
\end{aligned}
$$

$$
\begin{aligned}
f_3^{11} = \; & ((p_{31}+p_{32}+p_{34})((p_{14}p_{23}p_{32}p_{41}-p_{13}p_{24}p_{31}p_{42})(-p_{13}p_{24}p_{42}+p_{14}(p_{21}p_{41}+p_{23}p_{41}+p_{24}p_{41}-p_{24}p_{42})) \\
& +p_{12}^2p_{21}p_{34}p_{43}(-p_{24}p_{42}+p_{21}(p_{41}+p_{42}+p_{43}))-p_{12}(p_{13}p_{24}p_{42}(-p_{24}p_{31}p_{42}+ \\
& p_{21}(p_{34}p_{43}+p_{31}(p_{41}+p_{42}+p_{43})))+p_{14}(p_{23}p_{24}p_{32}p_{41}p_{42}+p_{21}^2p_{34}p_{41}p_{43}+ \\
& p_{21}(p_{24}(-2p_{31}p_{41}p_{42}-2p_{32}p_{41}p_{42}-2p_{34}p_{41}p_{42}+p_{34}p_{41}p_{43}+p_{34}p_{42}p_{43}) \\
& +p_{23}p_{41}(p_{34}p_{43}+p_{32}(p_{41}+p_{42}+p_{43})))))))/D_{11}
\end{aligned}
$$

$$
\begin{aligned}
f_4^{11} = \; & ((p_{41}+p_{42}+p_{43})((-p_{14}p_{23}p_{32}+p_{13}(p_{21}p_{31}+p_{23}p_{31}+p_{24}p_{31}-p_{23}p_{32}))(-p_{14}p_{23}p_{32}p_{41}+ \\
& p_{13}p_{24}p_{31}p_{42}) \\
& +p_{12}^2p_{21}p_{34}(-p_{23}p_{32}+p_{21}(p_{31}+p_{32}+p_{34}))p_{43}-p_{12}(p_{14}p_{23}p_{32}(-p_{23}p_{32}p_{42}+ \\
& p_{21}(p_{31}p_{42}+p_{32}p_{41}+p_{34}(p_{41}+p_{43})))+p_{13}(p_{23}p_{24}p_{31}p_{32}p_{42}+p_{21}^2p_{31}p_{34}p_{43}+ \\
& p_{21}(p_{24}p_{31}(p_{31}p_{42}+p_{32}p_{42}+p_{34}(p_{42}+p_{43}))+p_{23}(p_{32}p_{34}p_{43}+ \\
& p_{31}(p_{34}p_{43}-2p_{32}(p_{41}+p_{42}+p_{43}))))))))/D_{11}
\end{aligned}
$$

and,

$$
\begin{aligned}
D_7 \;=\;& ((p_{14}p_{23}p_{32}-p_{13}(p_{21}p_{31}+p_{23}p_{31}+p_{24}p_{31}-p_{23}p_{32}))^2+\\
& p_{12}^2(p_{23}p_{32}-p_{21}(p_{31}+p_{32}+p_{34}))^2-2p_{12}(p_{14}p_{23}p_{32}(-p_{23}p_{32}+p_{21}(p_{31}+p_{32}+p_{34}))+\\
& p_{13}(p_{23}(p_{24}p_{31}+p_{23}(p_{31}-p_{32}))p_{32}+p_{21}^2p_{31}(p_{31}+p_{32}+p_{34})+\\
& p_{21}(p_{24}p_{31}(p_{31}+p_{32}+p_{34})+p_{23}(p_{31}^2+p_{32}(p_{32}+p_{34})+p_{31}(3p_{32}+p_{34}))))))
\end{aligned}
$$

$$
\begin{aligned}
D_8 \;=\;& ((p_{13}p_{24}p_{42}-p_{14}(p_{21}p_{41}+p_{23}p_{41}+p_{24}p_{41}-p_{24}p_{42}))^2+\\
& p_{12}^2(p_{24}p_{42}-p_{21}(p_{41}+p_{42}+p_{43}))^2-2p_{12}(p_{13}p_{24}p_{42}(-p_{24}p_{42}+p_{21}(p_{41}+p_{42}+p_{43}))+\\
& p_{14}(p_{24}(p_{23}p_{41}+p_{24}(p_{41}-p_{42}))p_{42}+p_{21}^2p_{41}(p_{41}+p_{42}+p_{43})+\\
& p_{21}(p_{23}p_{41}(p_{41}+p_{42}+p_{43})+p_{24}(p_{41}^2+p_{42}(p_{42}+p_{43})+p_{41}(3p_{42}+p_{43}))))))
\end{aligned}
$$

$$
\begin{aligned}
D_9 \;=\;& (p_{13}^2(p_{34}p_{43}-p_{31}(p_{41}+p_{42}+p_{43}))^2+\\
& (p_{12}p_{34}p_{43}-p_{14}(p_{31}p_{41}+p_{32}p_{41}+p_{34}p_{41}-p_{34}p_{43}))^2-\\
& 2p_{13}(p_{12}p_{34}p_{43}(-p_{34}p_{43}+p_{31}(p_{41}+p_{42}+p_{43}))+p_{14}(p_{34}(p_{32}p_{41}+p_{34}(p_{41}-p_{43}))p_{41}+\\
& p_{31}^2p_{41}(p_{41}+p_{42}+p_{43})+p_{31}(p_{32}p_{41}(p_{41}+p_{42}+p_{43})+p_{34}(p_{41}^2+p_{43}(p_{42}+p_{43})+p_{41}(p_{42}+3p_{43}))))))
\end{aligned}
$$

$$
\begin{aligned}
D_{10} \;=\; & (p_{23}^2(p_{34}p_{43}-p_{32}(p_{41}+p_{42}+p_{43}))^2+\\
& (p_{21}p_{34}p_{43}-p_{24}(p_{31}p_{42}+p_{32}p_{42}+p_{34}p_{42}-p_{34}p_{43}))^2-\\
& 2p_{23}(p_{21}p_{34}p_{43}(-p_{34}p_{43}+p_{32}(p_{41}+p_{42}+p_{43}))+\\
& p_{24}(p_{34}^2(p_{42}-p_{43})p_{43}+p_{32}^2p_{42}(p_{41}+p_{42}+p_{43})+\\
& p_{32}p_{34}(p_{42}^2+3p_{42}p_{43}+p_{43}^2+p_{41}(p_{42}+p_{43}))+p_{31}p_{42}(p_{34}p_{43}+p_{32}(p_{41}+p_{42}+p_{43})))))
\end{aligned}
$$

$$D_{11} = (2(p_{14}^2 p_{23} p_{32}(p_{21} p_{41}(p_{31} p_{41} + p_{32} p_{41} + p_{34}(p_{41} - p_{43})) + p_{24}(p_{41} - p_{42})(p_{31} p_{41} +$$

$$p_{32} p_{41} + p_{34}(p_{41} - p_{43})) + p_{23} p_{41}(p_{31} p_{41} + p_{34}(p_{41} - p_{43}) +$$

$$p_{32}(2p_{41} + p_{42} + p_{43}))) + p_{13}^2 p_{24} p_{42}(p_{21} p_{31}(-p_{34} p_{43} +$$

$$p_{31}(p_{41} + p_{42} + p_{43})) + p_{23}(p_{31} - p_{32})(-p_{34} p_{43} + p_{31}(p_{41} +$$

$$p_{42} + p_{43})) + p_{24} p_{31}(p_{32} p_{42} + p_{34} p_{42} - p_{34} p_{43} +$$

$$p_{31}(p_{41} + 2p_{42} + p_{43}))) + p_{12}^2 p_{34} p_{43}(p_{23} p_{24} p_{32} p_{42} +$$

$$p21^2(p31(p41 + p42 + p43) + p32(p41 + p42 + p43) + p34(p41 +$$

$$p42 + 2p43)) - p21(p24(p31p42 + p32p42 + p34p42 - p34p43) +$$

$$p23(-p34p43 + p32(p41 + p42 + p43)))) + p13p14(-p24^2 p31(p41 - p42)(p31p42 +$$

$$p32p42 + p34(p42 - p43)) + p21^2 p31p34p41p43 - p23^2(p31 - p32)p41(-p34p43 +$$

$$p32(p41 + p42 + p43)) - p21(p24p31(p31p41p42 + p32p41p42 + p34p41p42 -$$

$$2p34p41p43 + p34p42p43) + p23p41(p32p34p43 + p31(-2p34p43 + p32(p41 +$$

$$p42 + p43)))) - p23p24(p31^2 p41p42 + p32(p32p41p42 + p34(-2p42p43 +$$

$$p41(p42 + p43))) + p31(p34(p41(p42 - 2p43) + p42p43) + p32(p41^2 +$$

$$p42(p42 + p43) + p41(4p42 + p43))))) - p12(p14(p21^2 p34(p31p41 + p32p41 + p34(p41 -$$

$$p43))p43 + p23p32(p24(p31p41p42 + p32p41p42 + p34p41p42 + p34p41p43 -$$

$$2p34p42p43) - p23p41(-p34p43 + p32(p41 + p42 + p43))) + p21(-p24(p31p41 + p32p41 +$$

$$p_{32} p_{42} + p_{34}(p_{42} - p_{43})) + p_{23}(p34^2(p_{41} - p_{43})p_{43} + p34(p41 - p43))(p31p42 + p32^2 p41(p41 + p42 + p43) +$$

$$p_{31} p_{41}(p_{34} p_{43} + p_{32}(p_{41} + p_{42} + p_{43})) + p_{32} p_{34}(p_{41}^2 + p_{43}(p_{42} + p_{43}) + p_{41}(p_{42} + 4p_{43}))))) +$$

$$p_{13}(p_{21}^2 p_{34} p_{43}(-p_{34} p_{43} + p_{31}(p_{41} + p_{42} + p_{43})) + p_{24} p_{42}(-p_{24} p_{31}(p_{31} p_{42} + p_{32} p_{42} +$$

$$p_{34} p_{42} - p_{34} p_{43}) + p_{23}(-2p_{32} p_{34} p_{43} + p_{31}(p_{34} p_{43} + p_{32}(p_{41} + p_{42} + p_{43})))) +$$

$$p_{21}(-p_{23}(-p_{34} p_{43} + p_{31}(p_{41} + p_{42} + p_{43}))(-p_{34} p_{43} + p_{32}(p_{41} + p_{42} + p_{43})) + p_{24}(p_{34}(p_{32} p_{42} +$$

$$p_{34}(p_{42} - p_{43}))p_{43} + p_{31}^2 p_{42}(p_{41} + p_{42} + p_{43}) + p_{31}(p_{32} p_{42}(p_{41} + p_{42} + p_{43}) + p_{34}(p_{42}^2 + 4p_{42} p_{43} + p_{43}^2$$

$$+ p_{41}(p_{42} + p_{43})))))))))$$

It is evident that the mating dynamics become significantly more complex with four mating types, and so a rigorous stability analysis becomes challenging. We analyzed the stability of E1 under the simplifying assumption of pairwise symmetric preferences/interactions between mating types. That is, we assume that $p_{12} = p_{21} = \gamma$, $p_{13} = p_{23} = \gamma + \eta$, $p_{14} = p_{41} = \gamma + \kappa$, $p_{23} = p_{32} = \gamma + \lambda$, $p_{24} = p_{42} = \gamma + \mu$ and $p_{34} = p_{43} = \gamma + \nu$. This allowed us to solve for the eigenvalues of the system described by equations for $f_1 - f_4$ which are given by,

$$\lambda_{1,2} = 0$$

$$\lambda_3 = \frac{6\gamma^3 + \eta\mu^2 + \kappa\mu^2 + \kappa^2(\lambda+\mu) + \gamma^2(\eta + 7\kappa + \lambda + 7\mu) + \gamma(3\kappa^2 + 2\kappa(\lambda + 2\mu) + \mu(2\eta + 3\mu))}{\gamma^2(3\gamma + \kappa + \mu + \nu)}$$

$$\lambda_4 = \frac{(6\gamma^3 + \eta\lambda^2 + \kappa\lambda^2 + \eta^2(\lambda+\mu) + \gamma^2(7\eta + \kappa + 7\lambda + \mu) + \gamma(3\eta^2 + \lambda(2\kappa + 3\lambda) + 2\eta(2\lambda + \mu)))}{\gamma^2(3\gamma + \eta + \lambda + \nu)}$$

Dividing through by γ^3 the expressions for λ_3 and λ_4 can be rewritten as,

$$\lambda_3 = \frac{6 + \tilde{\eta}\tilde{\mu}^2 + \tilde{\kappa}\tilde{\mu}^2 + \tilde{\kappa}^2(\tilde{\lambda}+\tilde{\mu}) + (\tilde{\eta} + 7\tilde{\kappa} + \tilde{\lambda} + 7\tilde{\mu}) + (3\tilde{\kappa}^2 + 2\tilde{\kappa}(\tilde{\lambda} + 2\tilde{\mu}) + \tilde{\mu}(2\tilde{\eta} + 3\tilde{\mu}))}{3 + \tilde{\kappa} + \tilde{\mu} + \tilde{\nu}}$$

$$\lambda_4 = \frac{6 + \tilde{\eta}\tilde{\lambda}^2 + \tilde{\kappa}\tilde{\lambda}^2 + \tilde{\eta}^2(\tilde{\lambda}+\tilde{\mu}) + (\tilde{\kappa} + 7\tilde{\eta} + 7\tilde{\lambda} + \tilde{\mu}) + (3\tilde{\eta}^2 + \tilde{\lambda}(2\tilde{\kappa} + 3\tilde{\lambda}) + 2\tilde{\eta}(2\tilde{\lambda} + \tilde{\mu}))}{3 + \tilde{\eta} + \tilde{\lambda} + \tilde{\nu}}$$

where $\tilde{\eta} = \frac{\eta}{\gamma}$, $\tilde{\kappa} = \frac{\kappa}{\gamma}$, $\tilde{\lambda} = \frac{\lambda}{\gamma}$ and so on. It follows that E1 is stable if,

$$\frac{6 + \tilde{\eta}\tilde{\mu}^2 + \tilde{\kappa}\tilde{\mu}^2 + \tilde{\kappa}^2(\tilde{\lambda}+\tilde{\mu}) + (\tilde{\eta} + 7\tilde{\kappa} + \tilde{\lambda} + 7\tilde{\mu}) + (3\tilde{\kappa}^2 + 2\tilde{\kappa}(\tilde{\lambda} + 2\tilde{\mu}) + \tilde{\mu}(2\tilde{\eta} + 3\tilde{\mu}))}{3 + \tilde{\kappa} + \tilde{\mu} + \tilde{\nu}} < 1$$

and

$$\frac{6 + \tilde{\eta}\tilde{\lambda}^2 + \tilde{\kappa}\tilde{\lambda}^2 + \tilde{\eta}^2(\tilde{\lambda}+\tilde{\mu}) + (\tilde{\kappa} + 7\tilde{\eta} + 7\tilde{\lambda} + \tilde{\mu}) + (3\tilde{\eta}^2 + \tilde{\lambda}(2\tilde{\kappa} + 3\tilde{\lambda}) + 2\tilde{\eta}(2\tilde{\lambda} + \tilde{\mu}))}{3 + \tilde{\eta} + \tilde{\lambda} + \tilde{\nu}} < 1$$

Although it is clear that the stability of E_1 is contingent upon the relationship between the different p_{ij}

values, the expressions above provides a far from intuitive justification for the stability. In other words, the increase in the dimensionality of the system makes an analytical treatment hard to interpret. Instead we use numerical simulations to illustrate the evolution of the system from multiple to two mating types and vice versa, for specific values of p_{ij}. For example, Fig. S2a shows a case where a population of four mating types evolves to only two mating types. In this case, p_{12} and p_{21} were set to significantly higher values than all other p_{ij}. Hence, mating types 1 and 2 become more likely to mate with each other than any other type which results to the elimination of types 2 and 3. In Fig. S2 a similar situation is assumed but now the increase of p_{12} and p_{12} relative to all other p_{ij} values is smaller. This leads to four mating types persisting in the population but now mating types 1 and 2 have slightly frequencies. We speculate that such small differences in p_{ij} values and subsequent increase in the frequencies of mating types that more frequently meet each other could lead to coevolution amongst those types, a subsequent increase in their mutual p_{ij} values and eventually to the evolution of only two mating types. Fig. S2b shows a situation where four mating types evolve to three. The conditions required for the reduction from four to three mating types are more strict than those required to evolve from four to three mating types. Finally, Fig. S2d shows the evolution from two to four mating types when all mating types interact equally with one another.

We used Mathematica 10.2.0.0 to run simulations and to generate all model figures in the MS and to simplify algebraic expressions.

3 Supplemental Figures

Fig.S1 Evolution of mating type frequencies assuming four possible mating types ($n = 3$). The frequencies of the three mating types are iterated over time using Equations (2 - 4) in the main text. Values for p_{ij} used a: $p_{12} = p_{21} = p_{31} = p_{31} = 1$, $p_{13} = p_{23} = 0.6$ for all other pairs (i, j). This choice of parameters leads to the loss of one of the three mating types. Note that all three mating types start at equal frequencies ($f_i = 1/3$ for all i), but mating type 3 is eliminated (green line). Mating types 1 and 2 follow the same trajectories to reach frequency 1/2.

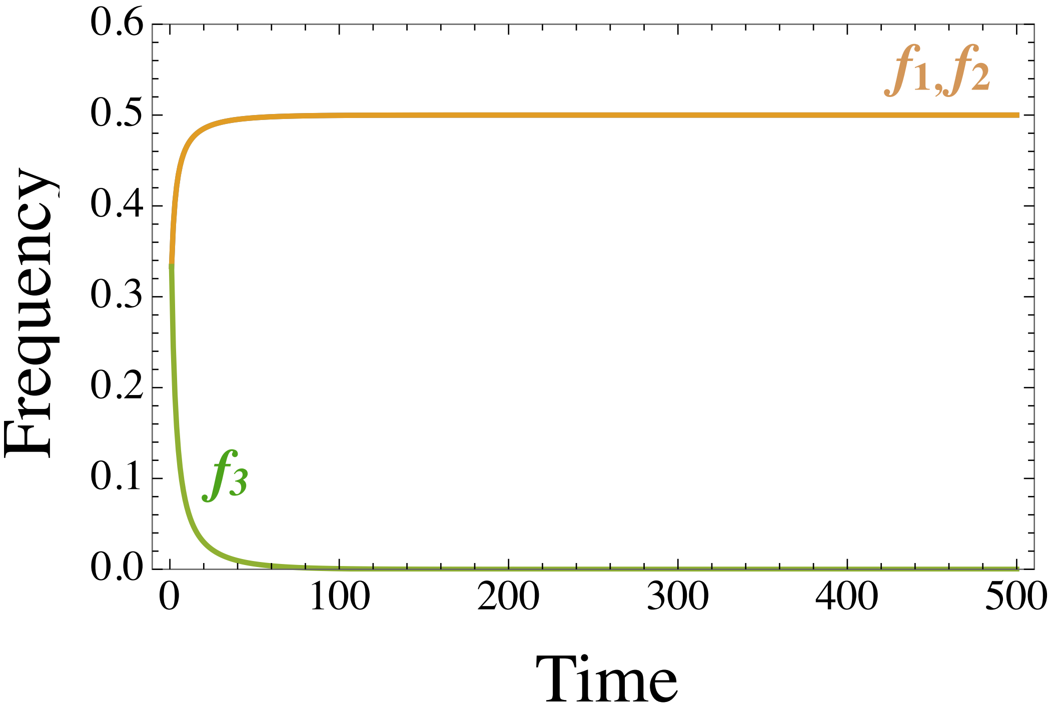

f_1,f_2
f_3
Frequency
Time
0.0
0.1
0.2
0.3
0.4
0.5
0.6
0
100
200
300
400
500

Fig.S2 Evolution of mating type frequencies assuming four possible mating types (n = 4). Values for p_{ij} used a: p_{12} = 1.9, p_{21} = 1.5, p_{ij} = 1 for all other pairs (i, j), b: p_{12} = 1.1 = p_{21}, p_{ij} = 1 for all other pairs (i, j), c: $p_{12} = p_{13} = p_{21} = p_{23} = p_{31} = p_{32}$ = 1.5, p_{ij}= 1 for all other (i, j) and d:p_{ij} = 1 for all (i, j).

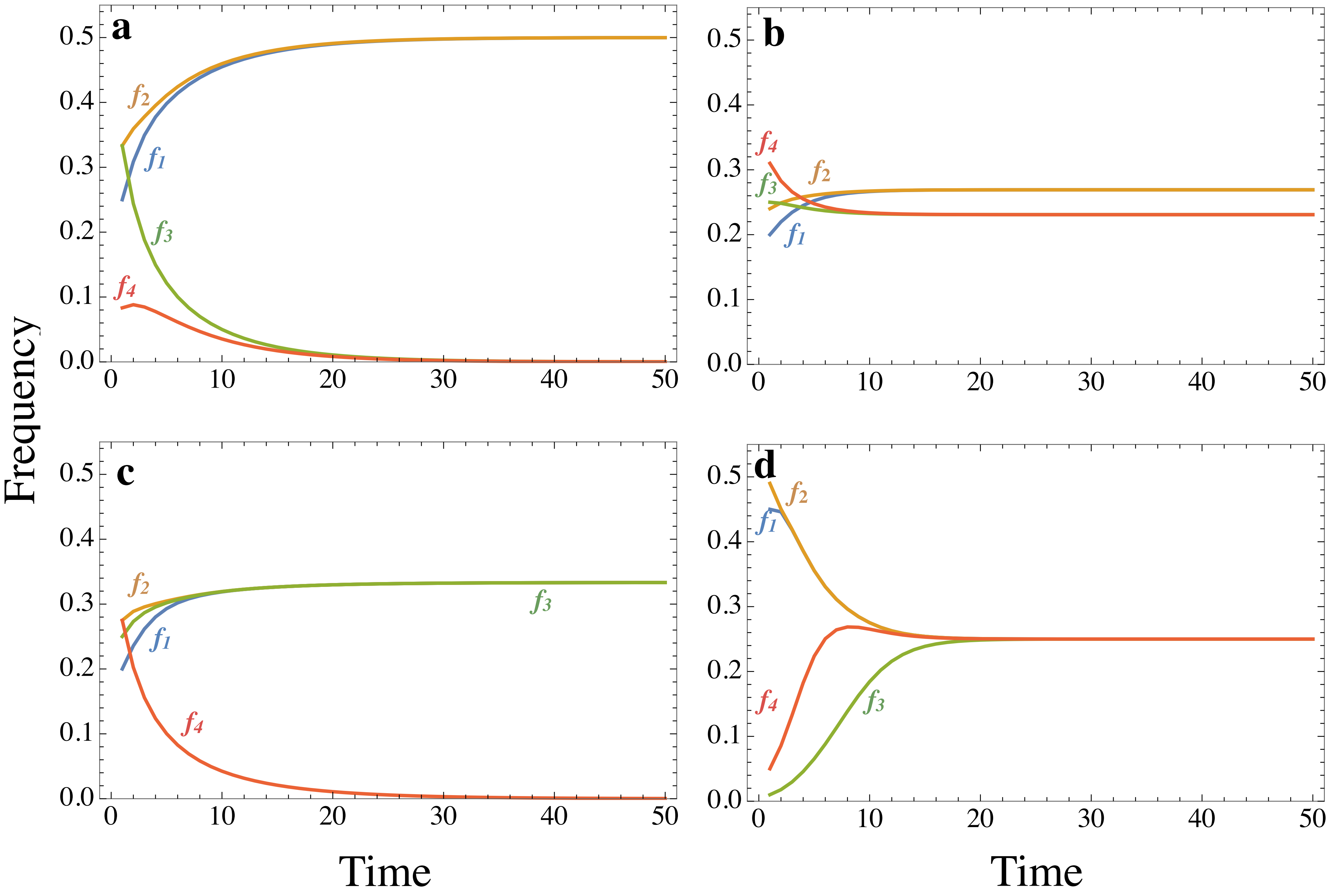
a
b
c
d
f2
f1
f3
f4
Frequency
Time
Time
0.0
0.1
0.2
0.3
0.4
0.5
0
10
20
30
40
50
